# The Most Popular Smartphone Apps for Weight Loss: A Quality Assessment

**DOI:** 10.2196/mhealth.4334

**Published:** 2015-12-16

**Authors:** Juliana Chen, Janet E Cade, Margaret Allman-Farinelli

**Affiliations:** ^1^ School of Molecular Bioscience Charles Perkins Centre University of Sydney Camperdown Australia; ^2^ Nutritional Epidemiology Group School of Food Science and Nutrition University of Leeds Leeds United Kingdom

**Keywords:** behavior change techniques, evaluation, obesity, quality, smartphone apps, weight management

## Abstract

**Background:**

Advancements in mobile phone technology have led to the development of smartphones with the capability to run apps. The availability of a plethora of health- and fitness-related smartphone apps has the potential, both on a clinical and public health level, to facilitate healthy behavior change and weight management. However, current top-rated apps in this area have not been extensively evaluated in terms of scientific quality and behavioral theory evidence base.

**Objective:**

The purpose of this study was to evaluate the quality of the most popular dietary weight-loss smartphone apps on the commercial market using comprehensive quality assessment criteria, and to quantify the behavior change techniques (BCTs) incorporated.

**Methods:**

The top 200-rated Health & Fitness category apps from the free and paid sections of Google Play and iTunes App Store in Australia (n=800) were screened in August 2014. To be included in further analysis, an app had to focus on weight management, include a facility to record diet intake (self-monitoring), and be in English. One researcher downloaded and used the eligible apps thoroughly for 5 days and assessed the apps against quality assessment criteria which included the following domains: accountability, scientific coverage and content accuracy of information relevant to weight management, technology-enhanced features, usability, and incorporation of BCTs. For inter-rater reliability purposes, a second assessor provided ratings on 30% of the apps. The accuracy of app energy intake calculations was further investigated by comparison with results from a 3-day weighed food record (WFR).

**Results:**

Across the eligible apps reviewed (n=28), only 1 app (4%) received full marks for accountability. Overall, apps included an average of 5.1 (SD 2.3) out of 14 technology-enhanced features, and received a mean score of 13.5 (SD 3.7) out of 20 for usability. The majority of apps provided estimated energy requirements (24/28, 86%) and used a food database to calculate energy intake (21/28, 75%). When compared against the WFR, the mean absolute energy difference of apps which featured energy intake calculations (23/28, 82%) was 127 kJ (95% CI -45 to 299). An average of 6.3 (SD 3.7) of 26 BCTs were included.

**Conclusions:**

Overall, the most popular commercial apps for weight management are suboptimal in quality, given the inadequate scientific coverage and accuracy of weight-related information, and the relative absence of BCTs across the apps reviewed. With the limited regulatory oversight around the quality of these types of apps, this evaluation provides clinicians and consumers an informed view of the highest-quality apps in the current popular app pool appropriate for recommendation and uptake. Further research is necessary to assess the effectiveness of apps for weight management.

## Introduction

Obesity is an accelerating global health challenge. The Global Burden of Disease Study 2013 reports 37% of adults (2.1 billion) globally are overweight or obese, with a prevalence of more than 60% in Australia, the United Kingdom, and the United States [1]. Given the magnitude of the epidemic, treatment strategies and interventions with long-term effectiveness and a wide reach are required to address this major public health concern.

Among researchers, there is growing interest into the use of smartphones to deliver behavioral interventions for health because of their cost advantages, ubiquity, and portability [2,3]. Estimates of adult smartphone ownership are 64%, 54%, and 51% in Australia [4], the United Kingdom, and the United States, respectively [5]. A total of 68% of Australian [6], 86% of British [7], and 35% of American smartphone users [8] report downloading a smartphone app. Simultaneously, a plethora of health- and fitness-related apps are now available to individuals through the commercial market (eg, the Google Play store and iTunes App Store) [3,6] and their popularity is ever increasing [9].

Smartphone apps hold promise in supporting health behavior change and weight management [2,3,10-13]. To ensure that these apps are able to influence sustained positive health outcomes, quality assessment is necessary. A range of frameworks have been used to evaluate the quality of apps in a variety of medical and health promotion areas, such as cancer [14-16], diabetes [17,18], smoking [19-21], mental health [22-24], headaches [25], cardiology [26], alcohol [27,28], HIV [29], and pain management [30,31]. These evaluation frameworks include the analysis of content source and expertise, information quality, app technology and design, user engagement and ease of use, and behavioral theories.

Yet, despite the high prevalence of overweight and obesity, there are few evaluations of the quality of weight-management apps, such that even within a review and analysis of mobile health apps for the most prevalent conditions by the World Health Organization, there was no mention of evaluating apps addressing overweight and obesity [32]. To our knowledge, only two studies have evaluated the quality of weight-loss apps. One of the first studies to conduct a systematic analysis of smartphone and iPad weight-loss apps revealed that only eight of 54 apps were of good quality and less than a third had complete scientific accuracy of measurements and nutrition content linked to recommendations from evidence-based guidelines (eg, body mass index [BMI] and estimated energy requirements) [33]. Suboptimal information quality has also been found in Korean obesity-management smartphone apps [34].

When specifically considering the potential for commercial weight-loss apps to enable behavior change, the literature is similarly limited; however, there appears to be a shortage of evidence-based content [35,36] and behavioral theory-based strategies [13,37] being applied. Abraham and Michie’s [38] theory-linked taxonomy of behavior change techniques (BCTs) offers a method of assessing effective behavioral interventions by providing a systematic framework for categorizing the elements necessary for facilitating behavior change. This 26-item taxonomy has been used in a recent review, revealing that an average of 8.1 out of 26 BCTs were incorporated across 40 physical activity and dietary apps [39]. However, given that the majority were physical activity apps (n=30) and only six dietary apps were reviewed, a comprehensive analysis of the quality and evidence base of commercial dietary weight-loss apps, specifically, is warranted.

In addition to the scarcity of comparative studies assessing the quality and effectiveness of dietary weight-loss apps, the urgent and ongoing need for further evaluation of weight-loss apps is reinforced by the limited oversight and standardization around the quality of health and fitness apps by regulatory bodies, such as the US Food and Drug Administration (FDA) [40] and Australia’s Therapeutic Goods Administration [41]. Therefore, the aims of this study were to extend the body of research assessing the quality of such apps by, firstly, examining the accountability, scientific coverage, accuracy, technology-enhanced features, and usability of popular dietary weight-loss smartphone apps and, secondly, by quantifying BCT incorporation in these apps.

## Methods

### Sample Attainment

Dietary and weight-loss smartphone apps were located in the *Health & Fitness* category of the Google Play store and iTunes App Store in Australia on August 15, 2014. Using a method from previous studies which determined popularity [35-37], the first 200 ranked apps in the *Top Selling* and *Top Apps* or *Top Paid Apps* and *Top Free Apps* sections of the respective stores above were selected.

Each app underwent initial screening based on the descriptions and associated screenshot images provided by the stores. Inclusion in this evaluation required that the app meet the following criteria: (1) was intended for weight management, (2) addressed dietary behaviors, (3) involved the tracking of energy intake, nutrients, or foods, as self-monitoring has been found to have a consistent association with weight loss, both in intervention programs [42] as well as in smartphone-based strategies [2], (4) had stand-alone functionality (ie, not requiring subscription to another program to operate) [37], and (5) was in English. Apps which were miscategorized under the *Health & Fitness* category or that addressed other health behaviors were excluded. Specific diet subcategory (eg, paleo diet) apps were also excluded because of limited generalizability [37].

Apps which fulfilled the inclusion criteria were downloaded onto a Samsung S2 smartphone running Jelly Bean 4.1.2 software (for Google Play store Android apps) and onto an iPhone 5 running iOS 7.0.1 (for Apple apps). After one day of use of the app, another screening against the inclusion criteria was undertaken, and those failing to meet the criteria were excluded. Duplicate apps which were available on both the Android and iPhone platforms were selected for use only on the Android operating system.

### Evaluation Criteria

As no widely accepted standards of quality evaluation for apps existed, a pro forma evaluation based on a modified version of the instrument developed by Gan and Allman-Farinelli [33] was collaboratively developed between the University of Sydney, Australia, and the University of Leeds, the United Kingdom. The tool contained the following basic descriptive information: name of app, developer, version, number of downloads, average ratings (ie, average number of stars that the app was rated), total number of ratings, number of users who voluntarily rated it, and price. The tool also included the following quality assessment features: accountability, scientific coverage and content accuracy, technology-enhanced features, usability, and incorporation of BCTs (see Table 1). Accountability measures, based on Silberg’s standards [43], evaluated an app’s authorship (ie, the author’s credentials and affiliations), attribution (ie, provision of information sources and references), disclosure (ie, sponsorship disclosure), and currency (ie, how up-to-date the content was kept).

Scientific coverage and content accuracy examined the range and accuracy of information related to weight management and general dietary advice provided by the apps. The practice guidelines for the treatment and management of overweight and obesity in adults released by the Dietitians Association of Australia (DAA) [44] and the National Health and Medical Research Council (NHMRC) [45] were consulted to determine the elements that would be relevant to a weight-management app based on a self-management approach. The features of healthy eating were derived from the NHMRC Australian Dietary Guidelines [46] and Nutrient Reference Values (NRVs) [46].

An additional element was included to further determine the accuracy of these dietary weight-loss apps in assessing energy intake. A weighed food record (WFR), which is considered to be the gold standard of dietary assessment [47], was kept by the first author (JC) for 3 days. The same 3-days’ worth of food intake was entered into all the apps, but using household measures or the default serving sizes provided by the app so as to mimic the food tracking process which would be conducted by a normal user. To determine the accuracy of the energy intake values provided by the apps, they were compared with the WFR results that were analyzed using the nutrient analysis software package FoodWorks, version 7 (Xyris Software) [48] with the Australian Food and Nutrient Database (AUSNUT) 2007 [49].

Apps were also appraised for their inclusion of a range of 14 technology-enhanced features compiled from common features observed in previous app evaluations, as these have been reported to reduce burden or enhance engagement in behavioral strategies [35,36]. The usability of apps was measured by the validated 10-item System Usability Scale (SUS), where items are ranked using a 5-point Likert scale, giving an overall usability score of 0-100 [50,51], and matched with a 7-point adjective rating scale: *worst imaginable, awful, poor, ok, good, excellent,* or *best imaginable* [52].

Abraham and Michie’s [38] 26-item taxonomy presented in the following three-phase categorization format was used: (1) motivational enhancing, (2) planning and preparation, and (3) goal striving and persistence [53]. This categorization format was used as a framework to quantify the incorporation of 26 BCTs into the apps, as this taxonomy provides a systematic method of identifying effective behavior change elements.

A total composite score out of 100 for their fulfilment of the different features of the quality assessment evaluation criteria was given to each app. The scoring system awarded the highest weight to scientific coverage and accuracy (32 points) and BCTs (26 points), followed by usability (SUS score out of 100 was scaled down proportionally to 20 points), technology-enhanced features (14 points), and accountability (8 points).

**Table 1:** Quality assessment evaluation criteria and scoring system.

Evaluation criteria				Maximum score^a^
**26 behavior change techniques (out of 26)**				
	For each of the 26 behavior change techniques^b^			1
**Accountability (out of 8)**				
	Authors credited			1
	Author’s affiliation			1
	Author’s credentials			2
	Information sources/references given			2
	Sponsorship disclosed			1
	App modified in the last month			1
**Scientific coverage and accuracy (out of 32)**				
	**Anthropometric assessment**			
		**Body mass index (BMI)**		
			Formula: BMI calculated and its use defined	2
			Interpretation of BMI: cutoff point for risk and treatment indicated when cutoff point exceeded; indicates healthy weight range	4
		Safety net on maximal weight loss which can be achieved		2
	Energy requirement calculator (calculates basal metabolic rate, energy requirement, or deficit based on individual's age, gender, physical activity level, and weight-loss goal)			4
	**Calorie counter**			
		Contains food database that helps calculate energy intake		4
		Energy intake calculations of apps coincide with 3-day WFR^c^		10
	**Features of healthy eating**			
		Calculates intake of macronutrients		2
		Recommends servings for five main food groups as per the AGHE^d^		2
		Recommends intake or limits other nutrients (ie, saturated fat, fiber, salt, and sugar) as per the AGHE and NRVs^e^		2
**Technology-enhanced features (out of 14)**				
	Weight/energy intake progress graphs or charts			1
	Recipes			1
	Pictures of food			1
	Barcode scanner			1
	Online social support/networking components (eg, Twitter and Facebook)			1
	Internet website links			1
	Food databases that can be modified (ie, add new foods and remember favorite foods)			1
	Educational material			1
	Reminders to log meals			1
	Calendar			1
	Flags for lapses in dietary goal adherence			1
	Physical activity tracking device (eg, accelerometer)/connection to activity apps			1
	Tracking of negative thoughts/stress			1
	Ability to export data/details about meals/daily summaries			1
**Usability (weighted score out of 20)**				
	SUS items^f^			N/A^g^

^a^A total composite score out of a maximum of 100 is calculated from the summation of 5 individual quality criteria subscores.

^b^As per Dusseldorp et al’s [53] three-phase categorization of behavior change techniques list.

^c^WFR: weighed food record.

^d^AGHE: Australian Guide to Healthy Eating.

^e^NRV: Nutrient Reference Value.

^f^SUS: System Usability Scale, as per Brooke’s [51] System Usability Scale list.

^g^N/A: not applicable. Each SUS item is rated on a 5-point Likert scale from 1 (strongly disagree) to 5 (strongly agree). Note that scores for individual SUS items are not meaningful on their own. The SUS score is calculated using a formula, with a maximum of 100, but in this evaluation tool the SUS score has been weighted.

### Evaluation Procedure

The evaluation process was discussed among all the other authors to develop a systematic approach for conducting the assessment. All authors were present in the discussion of the assessment criteria, and for consultation on the scoring of individual apps. One expert assessor conducted all the app quality evaluations, with the second assessor reviewing 30% of the apps for inter-rater reliability purposes. Where discrepancies arose in assessors’ results, they were resolved by discussion and, when necessary, in consultation with a third assessor. Apps were used by the first author (JC) for a total of 5 days and scored against the quality assessment criteria. On the first day, the assessor familiarized herself with the menu and interface and thoroughly explored the different functions and key features of the app. Food logging was also completed by the primary assessor after each midmeal and main meal on the first, second, and third days. In order to maintain consistency across the 5 days, additional engagement with the app was based upon push notification prompts and reminders from the app in order to replicate the frequency of app engagement which is likely to be carried out by individuals in a naturalistic setting.

### Data Analysis

The different components of the pro forma evaluation were analyzed using descriptive statistics. The frequency, mean, standard deviation, and relative rankings of apps were determined based on each assessment criteria and by overall score. Two-way mixed intraclass correlation coefficients (ICCs) were determined for inter-rater reliability. Absolute and percentage differences of the mean energy intake values of the 3 days were calculated for each app against the WFR. The overall mean of differences and 95% CIs of the apps from the WFR were calculated. Linear regression analysis was used to determine the relationship between the rankings of the apps as per the app stores (ie, popularity) versus the quality assessment criteria. *P*<.05 was considered significant. Statistical analyses were conducted using IBM SPSS Statistics for Windows, version 22.0 (IBM Corp) [54].

## Results

### Sample Characteristics

**Figure 1:**
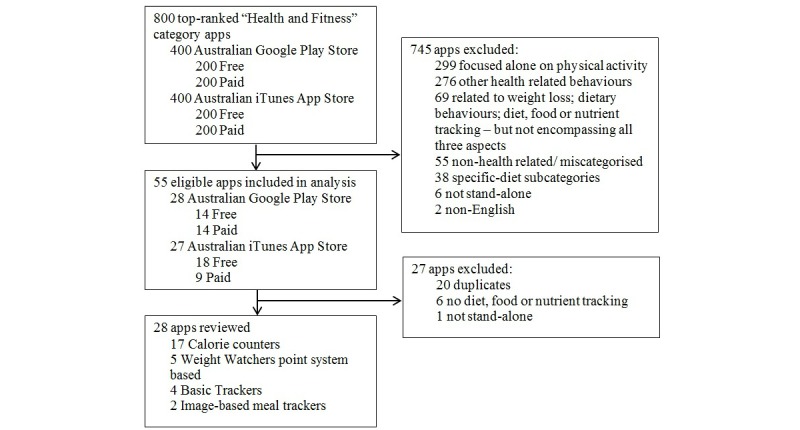
Flow diagram of the sampling procedure with the number of dietary weight-loss smartphone apps included or excluded.

From the sample of 800 top *Health & Fitness* category apps, 55 apps met the inclusion criteria following the initial screening and were downloaded to be included in the analysis. After the first day of use, 27 apps were excluded from further detailed evaluation because of duplication, lack of tracking functions, or not being stand-alone in functionality. A total of 28 apps were reviewed in detail, with 9 apps (32%) evaluated by both assessors. An excellent level of inter-rater reliability was observed (two-way mixed ICC .94, 95% CI .75-.99). These 28 apps were characterized into the following four categories: calorie counters (17/28, 61%), Weight Watchers point system-based apps (5/28, 18%), basic trackers which logged food or nutrients but contained no energy calculations (4/28, 14%), and image-based meal trackers which used a process of taking photos of meals in order to track intake rather than a focus on calorie counting (2/28, 7%) (see Figure 1).

A total of 23 apps out of 28 (82%) provided outputs which could be compared to the WFR and were included in the accuracy analysis of energy intake. The following 5 apps out of 28 (18%) were excluded from analysis as they did not calculate energy intake: Michelle Bridges 12 Week Body Transformation (12WBT)*;* Food Journal by Katie Wright; FoodTrackerPro by Aspyre Solutions; Argus—Pedometer, Run, Cycle; and TwoGrand.

### Quality of Apps

The scores for each of the evaluation components and the ranking of the apps based on their total quality score are summarized in Table 2. Noom Weight Loss Coach received the top ranking based on its overall score of 75. This was followed by Calorie Counter PRO by MyNetDiary and ControlMyWeight by CalorieKing (score of 65). The app receiving the lowest overall score was Food Journal by Katie Wright (score of 17). The mean overall score for the apps was 47.3 (SD 13.9).

**Figure 2:**
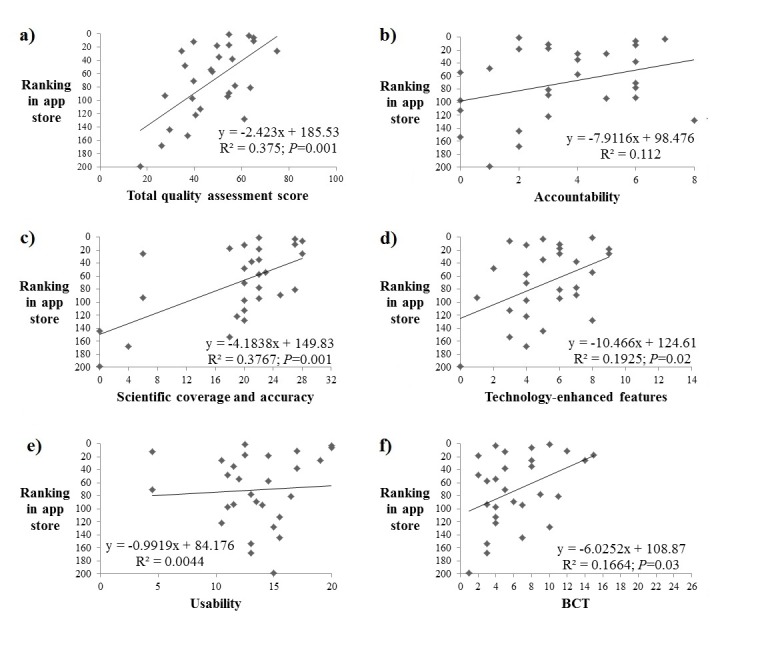
Regression analyses of the association between the rankings in the commercial app store (ie, popularity) versus quality assessment measures: (a) overall quality assessment score, (b) accountability, (c) scientific coverage and accuracy, (d) technology-enhanced features, (e) usability, and (f) behavior change technique (BCT). Note: ranking is numerical, with the rank of most popular apps starting from 1 and the least popular app ranked at 200.

Regression analyses determined that lower-numbered rankings in the app store (ie, greater popularity) were significantly associated with total quality assessment score (R^2^= .375; *P*=.001), scientific coverage and accuracy (R^2^=.377; *P*=.001), technology-enhanced features (R^2^=.192; *P*=.02), and incorporation of BCTs (R^2^=.166; *P*=.03), while showing no significant association with accountability and usability (see Figure 2).

### Accountability

Only 1 app out of 28 (4%), Calorie Counter by SparkPeople, fulfilled all the accountability criteria. The mean score for accountability was 3.5 out of 8 (SD 2.3; see Table 2). Over half the apps were modified within the last month (17/28, 61%) and credited the authors (16/28, 57%). Around 40% of the apps disclosed sponsorship (12/28, 43%), information sources and references for the food database used by the apps (12/28, 43%), and authors' affiliations (11/28, 39%). Under a third of the apps (9/28, 32%) reported authors or app development team members with scientific or health professional credentials.

### Scientific Coverage and Accuracy

**Figure 3:**
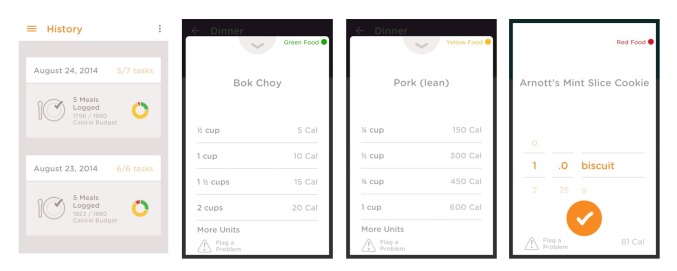
Screenshot of sample smartphone app which provides estimates of energy requirements and searchable food databases (from Noom Weight Loss Coach by Noom, Inc).

The mean score for scientific coverage and accuracy was 18.8 out of 32 (SD 8.0), with ControlMyWeight by CalorieKing and Noom Weight Loss Coach both receiving the highest scores (28 out of 32) (see Table 2). The majority of apps provided estimated energy requirements (24/28, 86%) and contained a food database that helped to calculate energy intake (21/28, 75%) (see Figure 3). Less than a third of apps incorporated each of the anthropometric assessment features—calculation of BMI and defining its use (9/28, 32%), interpretation of BMI and healthy weight range (8/28, 29%), and safety net on maximal weight loss (8/28, 29%). The different features of healthy eating were incorporated to different extents, with 16 apps out of 28 (57%) calculating intake of macronutrients. However, only 11 apps out of 28 (39%) recommended guidelines for achieving healthy dietary patterns, such as limiting saturated fat, salt, and sugar, and maximizing fiber intake. Only 6 of the 28 apps (21%) recommended servings for the five main food groups. TwoGrand and Food Journal by Katie Wright did not include any of the elements for scientific coverage and accuracy.

**Table 2:** Relative ranking of popular dietary weight-loss smartphone apps. Ranking was determined according to their total score which was calculated from the sum of the scores for each component of the quality assessment criteria.

Rank	App	Score					
		Acc.^a^	SCA^b^	TEF^c^	Us.^d^	BCT^e^	TS^f^
1^g^	Noom Weight Loss Coach by Noom, Inc (2010, USA)	5	28	9	19	14	75
2	Calorie Counter PRO by MyNetDiary, Inc (2010, USA)	3	27	6	17	12	65
2^g^	ControlMyWeight by CalorieKing Wellness Solutions (2012, Australia)	6	28	3	20	8	65
4	Food Diary and Calorie Tracker by MyNetDiary, Inc (2010, USA)	3	27	6	16.5	11	63.5
5^g^	Easy Diet Diary by Xyris Software (2011, Australia)	7	27	5	20	4	63
6	Calorie Counter by SparkPeople (2012, USA)	8	20	8	15	10	61
7^g^	Jillian Michaels Slim-Down: Weight Loss, Diet & Exercise Solution (2010, USA)	6	22	7	13	9	57
8	MyPlate Calorie Tracker LITE by Demand Media, Inc (2013, USA)	6	21	7	17	5	56
9^g^	Calorie Counter by MyFitnessPal, Inc (2009, USA)	2	22	8	12.5	10	54.5
9	Calorie Counter & Diet Tracker by Calorie Count (2010, USA)	3	25	7	13.5	6	54.5
9^g^	My Diet Coach Pro by InspiredApps (A.L) Ltd (2012, USA)	3	18	6	12.5	15	54.5
12	Nutritionist—Dieting made easy by Outlier (2011, USA)	5	22	6	14	7	54
13	My Diet Diary Calorie Counter by MedHelp, Inc (2011, USA)	4	22	5	11.5	8	50.5
14^g^	Calorie Counter by FatSecret (2010, USA)	2	22	9	14.5	2	49.5
15	Cronometer by BigCrunch Consulting, Ltd (2011, USA)	4	22	4	14.5	3	47.5
16	Value Diary Plus by Fenlander Software Solutions, Ltd (2011, UK)	0	23	8	12	4	47
17	Diet Watchers Diary by Croc Software (2012, Israel)	0	20	3	15.5	4	42.5
18	Body Tracker—body fat tracker by Linear Software, LLC (2012, N/A^h^)	3	19	4	10.5	4	40.5
19^g^	Map My (walk, run, ride, fitness) apps by MapMyFitness, Inc (2008, USA)	6	20	4	4.5	5	39.5
19	Map My + (walk, run, ride, fitness) apps by MapMyFitness, Inc (2008, USA)	6	20	4	4.5	5	39.5
21	Pts Plus Weight Diary by Frippware (2012, N/A)	0	20	4	11	4	39
22	Point Tracker Weight Watchers by PointTracker (N/A, UK)	0	18	3	13	3	37
23	Points Calculator & Weekly Weight Loss by Christian Robert Gossain (2011, N/A)	1	20	2	11	2	36
24	Argus—Pedometer, Run, Cycle by Azumio (2013, USA)	4	6	6	10.5	8	34.5
25	TwoGrand by TwoGrand, Inc (2013, USA)	2	0	5	15.5	7	29.5
26^g^	Michelle Bridges 12WBT^i^(2012, Australia)	6	6	1	11.5	3	27.5
27	FoodTrackerPro by Aspyre Solutions (2010, Australia)	2	4	4	13	3	26
28	Food Journal by Katie Wright (2012, USA)	1	0	0	15	1	17
Mean (SD)		3.5 (2.3)	18.8 (8.0)	5.1 (2.3)	13.5 (3.7)	6.3 (3.7)	47.3 (13.9)

^a^Acc.: accountability (out of 8).

^b^SCA: scientific coverage and accuracy (out of 32).

^c^TEF: technology-enhanced features (out of 14).

^d^Us.: usability (out of 20).

^e^BCT: behavior change technique (out of 26).

^f^TS: total score (out of 100).

^g^Evaluated by two assessors for inter-rater reliability purposes.

^h^N/A: not applicable (as country or year was unavailable).

^i^12WBT: 12 Week Body Transformation.

**Figure 4:**
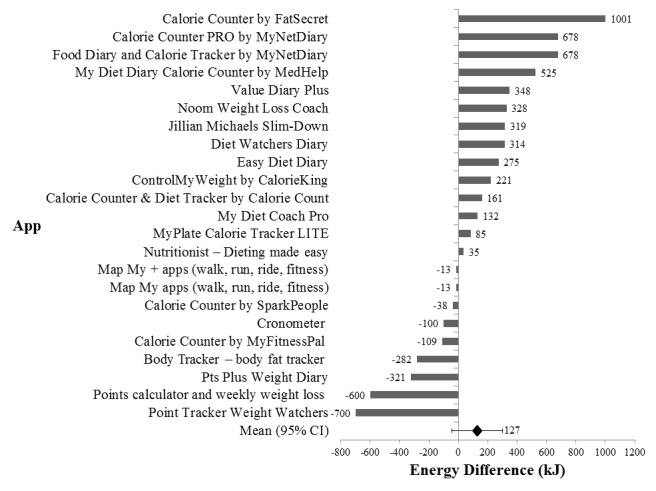
Accuracy of dietary apps compared to the weighed food record (WFR). Differences in mean energy intake values (kJ) over 3 days for dietary apps (n=23) were compared against the 3-day WFRs analyzed on FoodWorks. The overall mean difference of all the apps from the WFRs is denoted by the black diamond, and the 95% CI is indicated by the error bars.

Across the 23 apps which featured energy intake calculations, mean absolute energy difference when compared against the WFR was 127 kJ (95% CI -45 to 299) and mean percentage energy difference was 1.9% (95% CI -0.5 to 4.4; see Figure 4). Calorie Counter by FatSecret and Points Calculator & Weekly Weight Loss had the greatest discrepancy in reported energy intake values, with 1001 kJ (14%) greater and 700 kJ (10%) lower energy differences, respectively. In contrast, Map My (walk, run, ride, fitness) and Map My + (walk, run, ride, fitness) reported the smallest energy difference—13 kJ (0.2%) lower—when compared with the WFR.

### Technology-Enhanced Features

Out of the 14 technology-enhanced features considered, a mean of 5.1 features (SD 2.3) were identified across the apps. The apps with the greatest inclusion of technology-enhanced features were Calorie Counter by FatSecret and Noom Weight Loss Coach (score of 9 out of 14), while Food Journal by Katie Wright contained none of the features (see Table 2).

**Figure 5:**
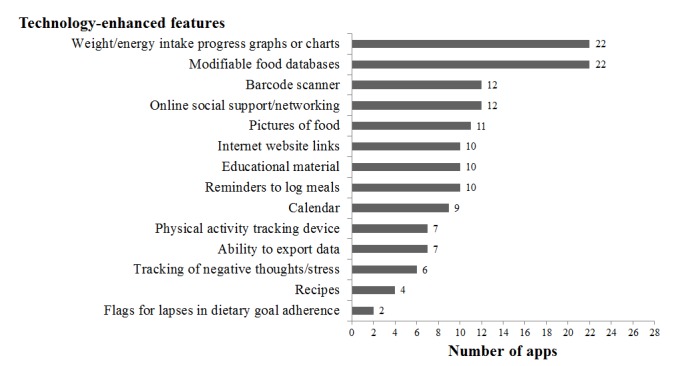
Incorporation of technology-enhanced features across apps. Number of total apps (n=28) incorporating each technology-enhanced feature.

**Figure 6:**
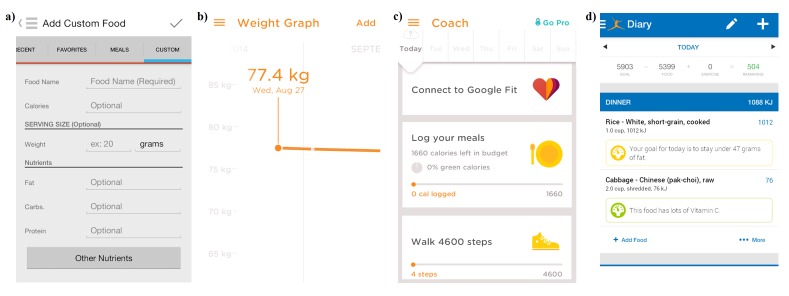
Screenshots of technology-enhanced features present in smartphone apps: (a) modifiable food database (from Calorie Counter by FatSecret), (b) weight progress charts (from Noom Weight Loss Coach by Noom, Inc), (c) built-in physical activity tracking device (eg, pedometer, accelerometer, or connection to other activity monitoring apps) (from Noom Weight Loss Coach by Noom, Inc), and (d) flags for lapses in dietary goal adherence (from MyFitnessPal by MyFitnessPal, Inc).

Weight or energy intake progress charts and modifiable food database were the most common technology-enhanced features present across the apps (22/28, 79%), followed by barcode scanners and online social support or networking, which were both included by 12 of the 28 apps (43%) (see Figure 5). A quarter of the apps (7/28, 25%) had the ability to export data, either for direct access by the individual or for a dietitian to access (eg, Easy Diet Diary), and included a built-in physical activity tracking device (eg, pedometer, accelerometer, or connection to other activity monitoring apps). Flags for lapses in dietary goal adherence was the least observed technology-enhanced feature, only appearing in 2 of the 28 apps (7%). Figure 6 illustrates examples of some of these features found in commercial apps.

### Usability

The mean SUS score was 67.5 (SD 18.5) out of 100 (range 0-100), equating to an adjective rating of *ok*. The majority of apps (25/28, 89%) had a usability rating from *ok* to *best imaginable*. The 2 apps out of 28 (7%) with the greatest usability scores were ControlMyWeight by CalorieKing and Easy Diet Diary by Xyris (see Table 2). These apps had a greater adaptability within the food database, allowing favorite, recent foods to be memorized, and had a range of household and metric measures, which increased the ease of self-monitoring food and energy intake. They also included additional features which fostered an increased engagement with the app.

### Incorporation of Behavior Change Techniques

An average of 6.3 (SD 3.7) of the 26 BCTs were included across the apps. The majority of the apps (26/28, 93%) integrated less than half of the BCTs. My Diet Coach Pro had the highest incorporation of BCTs (15 BCTs) followed by Noom Weight Loss Coach (14 BCTs), while Food Journal by Katie Wright had the lowest BCT inclusion (1 BCT) (see Table 2).

**Figure 7:**
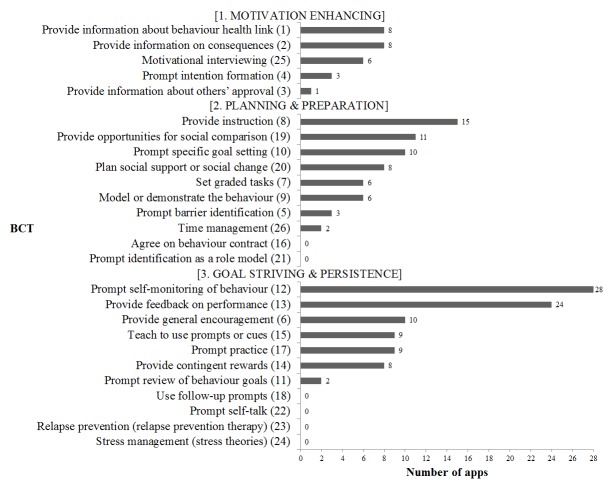
Incorporation of behavior change techniques (BCTs) across apps. Number of apps (n=28) incorporating each of the 26 individual BCTs according to the three-phase categories: motivation enhancing, planning and preparation, and goal striving and persistence.

Figure 7 highlights the proportion of apps that included each individual BCT, as per the three-phase categories. BCTs associated with goal striving and persistence were the most commonly incorporated, followed by planning and preparation, and lastly motivation enhancing. All apps incorporated *self-monitoring of behavior*, which predominantly appeared in the form of tracking of food or energy intake, and also through the monitoring of physical activity and exercise. *Feedback on performance* was also present in the majority of apps (24/28, 86%). Feedback predominantly appeared in the form of instant feedback, whereby energy intake was immediately updated when foods or exercises were logged. Longer-term trends of energy intake and weight progress were represented graphically by many apps. Only 2 apps out of 28 (7%)—Food Diary and Calorie Tracker, and Calorie Counter PRO—provided individualized and tailored feedback. Absent across all the apps were the following BCTs: agreeing on behavioral contract, identification of a role model, using follow-up prompts, self-talk, relapse prevention, and stress management (stress theories).

## Discussion

### Principal Findings

This evaluation indicates that, overall, the most popular dietary weight-loss apps available on the commercial market are suboptimal in quality. Few apps scored well for measures of accountability and while, overall, many apps scored reasonably in the domain of scientific coverage and accuracy, there was limited scientific coverage of information relevant to weight management. Although the agreement between apps and a dietitian-coded weighed food record was fair—mean difference 127 kJ, 95% CI -45 to 299—the accuracy of energy intake calculations was variable across different apps. There was restricted coverage of technology-enhanced features, and the usability of apps ranged from the *worst imaginable* to the *best imaginable*. A limited incorporation of BCTs was found across the apps reviewed.

Evaluations based on assessing the actual app content and the inclusion of evidence-based information have been determined to be better predictors of appraising the quality of apps than content-independent review methodologies [55]. Furthermore, the self-management nature of these commercial dietary weight-loss apps emphasizes the necessity of delivering robust and accurate evidence-based information through these apps to the consumer. Considering the extent of BCT incorporation also provides an indication of the likely effectiveness of these apps to facilitate behavior change [56,57].

The accountability of apps is fundamental, both for the public and for their use as weight management tools in clinical practice or for the distribution of wide-reaching public health interventions to target obesity. Although many apps were updated regularly, less than half of the reviewed apps disclosed sponsorship, provided references and sources of information, and declared the authors' affiliations, which parallels the poor accountability found in other studies of obesity-management apps, both in Australia [33] and Korea [34]. It is also disconcerting that there was an absence of scientific and health professionals guiding the design and development of these weight-loss apps. This may offer one explanation for the predominance of calorie counting apps and limited scientific coverage of information that would support weight management, such as energy balance, and foods and beverages to meet the requirements and recommendations from dietary guidelines. Lay users and dietitians have expressed similar sentiments over the credibility, comprehensiveness, accuracy, and general quality of health and fitness apps, as well as the reputability and legitimacy of the app sources [3,58]. This indicates a need for collaborative input from researchers and qualified health professionals to guide the improvement and development of comprehensive theory-based dietary weight-loss apps with tailored and targeted nutrition advice.

Furthermore, there was variability in the accuracy of energy intake calculations, although the mean difference was small with the 95% CI indicating nonstatistically significant variation between the two methods. However, some weight-management apps display a large discrepancy in values when compared to the WFR. For example, the second most highly ranked app—Calorie Counter PRO by MyNetDiary, Inc—also had the second highest energy overestimation at 678 kJ. These results highlight the range of potential under- or overestimation by apps; however, the results are concordant with other studies which have found that dietary assessment carried out by mobile phones and nutrition apps have similar validity and reliability [59] and moderate-to-good correlations for measuring energy and nutrition intakes [60] when compared with conventional methods. In this evaluation, even when a trained dietitian entered the foods and serving sizes, there were difficulties experienced due to the lack of alternative serving sizes or household measures, and the inability to match the foods consumed as the majority of apps used a US food database. Another source of variability in accuracy may be the modifiability of food databases in some apps, which although offers the benefit of user customization of foods consumed, nevertheless presents shortcomings in the accuracy of nutrients when users enter them in themselves, and also can lead to losses to the quality of the food database from alterations by crowdsourcing. Since tracking of energy intake is an important feature of many apps, the need for accuracy is important to avoid misleading the consumer.

Evidence suggests that weight loss is supported by frequent contact with an intervention [37,61]. However, in health and fitness apps, retention has been found to rapidly decrease from 47% retention at 30 days to only 30% at 90 days [62], and with a quarter of downloaded health apps only used once, and three-quarters discontinued after the tenth use [63]. Similarly, self-monitoring and use of a weight-loss app is reported to decline over a 6-month period [10,64]. Thus, the usability (ie, the efficiency, acceptability, and appeal) of an app for its target audience is critical, particularly if the aim is to use apps repeatedly to facilitate long-term changes in behavioral outcomes and weight loss. For the dietary weight-loss apps reviewed, the mean SUS of 67.5 is equivalent to an adjective rating of *ok*. However, the weight-loss apps best rated for usability in this review, such as ControlMyWeight by CalorieKing and Easy Diet Diary by Xyris, were rated *best imaginable* (SUS 100), and out-rated other apps for recording physical activity exertion (SUS 75.4; *good*) [65] and for diabetes self-management (SUS 84; *good*) [66]. Many of the dietary weight-loss apps reviewed would still benefit from improvements in the ease of app use, as well as user engagement, particularly if they are to be the medium for delivering public health or preventative health interventions for chronic disease and obesity management.

Food logging and self-monitoring can be burdensome and time-consuming, and can result in noncompliance and underestimation as usual dietary intakes may be altered to avoid the inconvenience of recording [67]. Hence, technology-enhanced features, such as barcode scanners, can assist in reducing user burden and in maintaining motivation and compliance for ongoing use of the apps through online social networking with health professionals and others trying to lose weight [2,35,36]. In this evaluation, the restricted range of technology-enhanced features integrated within commercial apps may be another contributor to the decline in engagement with an app, especially as the primary role of these weight-loss apps is to promote self-monitoring and tracking.

In Internet-based interventions, incorporation of more BCTs were found to have a larger effect on behavior than interventions with fewer techniques [57]. Across the 28 apps reviewed, less than a quarter of the 26 BCTs were included. This gap between the theoretical framework, which has established the potential to enable behavior change and weight loss, and its subsequent inclusion in apps is consistent with the findings from other evaluations of weight loss [13,35-37] and physical activity and dietary [39] apps. The hallmarks associated with effective healthy eating and physical activity interventions have been determined to be self-monitoring accompanied by at least one of the following: feedback on performance, intention formation, specific goal setting, and a review of behavioral goals [56]. All the apps analyzed in this review included self-monitoring of behavior, as it was an inclusion criterion of this evaluation, and feedback on performance was also present in the majority of apps. However, only 2 apps—My Diet Coach Pro and Noom Weight Loss Coach—included the range of BCTs commonly associated with greater effectiveness.

These 2 apps also included gamification, which is defined as using elements of game design in nongame contexts [68], and involves the inclusion of *motivational affordances*, such as points, levels, clear goals, feedback, rewards, progress, and challenges [69]. Gamification has been found to yield positive effects among a review of empirical studies on gamification [69]. Furthermore, in physical activity and dietary health and fitness apps, behavioral theory and, more specifically, motivational components of behavior were significantly associated with gamification [70]. This suggests that gamification not only has an apparent overlap with BCT constructs, but that it also has the potential to increase the motivation of app users in order to sustain habits and engage individuals with the behavioral strategies within apps through creation of positive, intrinsically motivating, “gameful” experiences [69,71,72]. Therefore, the gamification of weight-management apps may be a possible avenue for enhancing the delivery of behavioral theories, particularly motivational components; more in-depth study of the relationship between gamification and health behavior change is necessary.

One of the key strengths of this study was the theory-driven approach to app evaluation which allowed for a thorough examination of multiple parameters around the quality of the apps, such as accountability, scientific coverage, accuracy, technology-enhanced features, and usability, as well as behavioral theory. Particularly, as there is no industry standard or regulation of weight-management apps, this evaluation will assist health professionals in understanding which apps from among the current popular app pool are of the highest quality and appropriate for recommendation to their clients, and will assist in protecting consumers from misinformation.

### Limitations

It was not feasible in this study to evaluate all the weight-loss apps available to the public. Hence, it is possible that there are other apps that incorporate all the BCTs (but are not popular) or that some country-specific popular apps were missed. The accuracy of energy intake observed among all apps may have been influenced by the researcher being trained in nutrition (ie, a dietitian) and it is possible that the lay public would have more difficulty matching foods and interpreting the serving size to enter.

Although specific components of behavior change interventions may be understood to facilitate behavior change, they still require testing in trials. Emerging evidence suggests that caution should be exercised in the recommendation of apps in weight management. In randomized controlled trials evaluating commercial weight-loss smartphone apps such as MyFitnessPal [64] or Lose It! [73], although weight loss was observed in the intervention groups, the effects were not statistically significant when compared to the controls. In contrast, a researcher-developed weight-loss app, My Meal Mate (MMM), demonstrated significant weight reduction compared with controls in a 6-month period [10].

### Conclusions

With the relative absence of BCTs incorporated, along with variability in the additional measures of quality of these apps, such as the scientific coverage and accuracy, the recommendation of dietary weight-loss apps in clinical practice and in public health should proceed with caution. Ongoing evaluation of these apps and implementation of a standardized framework for quality assessment is necessary to drive the design and development of higher-quality apps on the market. Further research around efficacy trials of apps to promote weight loss is also warranted.

## Abbreviations

12WBT: 12 Week Body Transformation
Acc.: accountability
AGHE: Australian Guide to Healthy Eating
AUSNUT: Australian Food and Nutrient Database
BCT: behavior change technique
BMI: body mass index
DAA: Dietitians Association of Australia
FDA: Food and Drug Administration
ICC: intraclass correlation coefficient
N/A: not applicable
NHMRC: National Health and Medical Research Council
NRV: Nutrient Reference Value
SCA: scientific coverage and accuracy
SUS: System Usability Scale
TEF: technology-enhanced features
TS: total score
Us.: usability
WFR: weighed food record
